# Contemporary Transposon Tools: A Review and Guide through Mechanisms and Applications of *Sleeping Beauty*, *piggyBac* and *Tol2* for Genome Engineering

**DOI:** 10.3390/ijms22105084

**Published:** 2021-05-11

**Authors:** Nicolás Sandoval-Villegas, Wasifa Nurieva, Maximilian Amberger, Zoltán Ivics

**Affiliations:** Division of Medical Biotechnology, Paul Ehrlich Institute, 63225 Langen, Germany; Nicolas.SandovalVillegas@pei.de (N.S.-V.); Wasifa.Nurieva@pei.de (W.N.); Maximilian.Amberger@pei.de (M.A.)

**Keywords:** transposon, transposition, nonviral, genome engineering, transgenesis, induced pluripotent stem cells, gene therapy, genetic screens

## Abstract

Transposons are mobile genetic elements evolved to execute highly efficient integration of their genes into the genomes of their host cells. These natural DNA transfer vehicles have been harnessed as experimental tools for stably introducing a wide variety of foreign DNA sequences, including selectable marker genes, reporters, shRNA expression cassettes, mutagenic gene trap cassettes, and therapeutic gene constructs into the genomes of target cells in a regulated and highly efficient manner. Given that transposon components are typically supplied as naked nucleic acids (DNA and RNA) or recombinant protein, their use is simple, safe, and economically competitive. Thus, transposons enable several avenues for genome manipulations in vertebrates, including transgenesis for the generation of transgenic cells in tissue culture comprising the generation of pluripotent stem cells, the production of germline-transgenic animals for basic and applied research, forward genetic screens for functional gene annotation in model species and therapy of genetic disorders in humans. This review describes the molecular mechanisms involved in transposition reactions of the three most widely used transposon systems currently available (*Sleeping Beauty*, *piggyBac,* and *Tol2*), and discusses the various parameters and considerations pertinent to their experimental use, highlighting the state-of-the-art in transposon technology in diverse genetic applications.

## 1. Introduction

**Figure 1:**
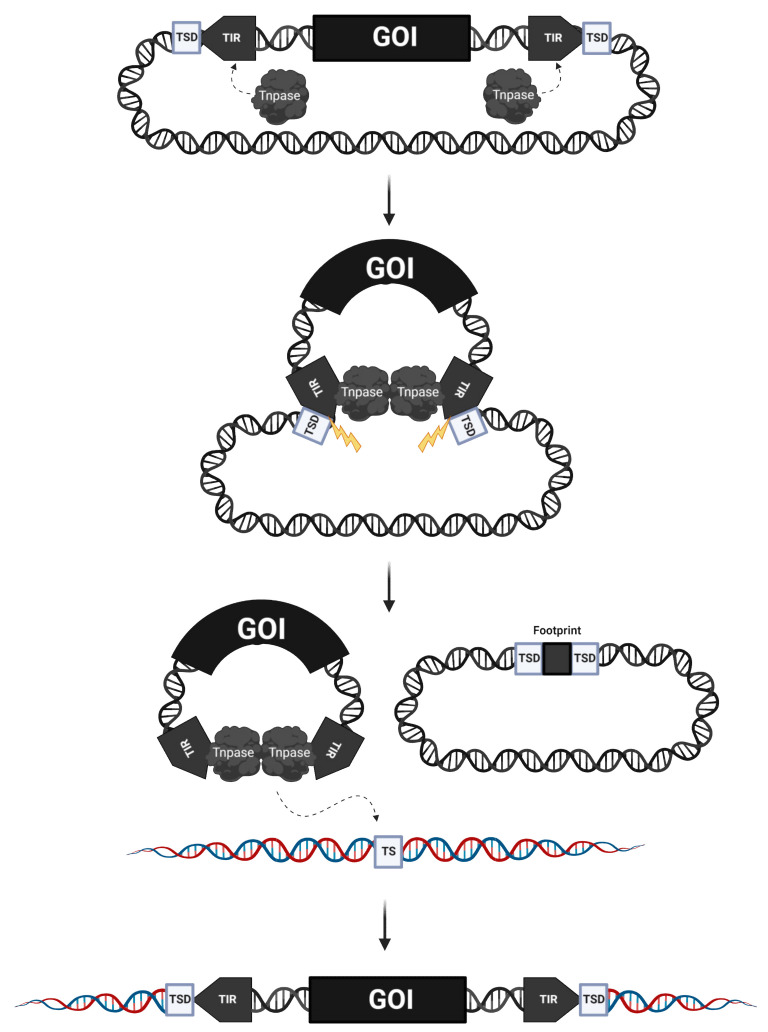
Schematic overview of transposase-mediated cut-and-paste transposition. A gene of interest (GOI, black bar) is mobilized by transposase (Tnpase) molecules (grey amorphous shape) from vector donor DNA (grey DNA stands) to a genomic locus (blue-red DNA strands). The transposase binds to the terminal inverted repeats (TIRs, grey arrows), induces double-stranded breaks (indicated with yellow lightning bolts), and excises the mobile element from the donor DNA leaving behind a footprint. The transposon-transposase complex finds a suitable target site (TS) and performs integration, producing a target site duplication (TSD).

Transposons are segments of DNA with the ability to change their positions within the genome. The most prominent mechanism of transposon movement is “cut-and-paste” transposition, during which a transposase enzyme mediates the excision of the element from its donor location and its reintegration into a new chromosomal locus (Figure 1). During transposition, the transposase (i) interacts with its binding sites in the terminal inverted repeats (TIRs) that define the boundaries of the transposon, (ii) promotes the assembly of a synaptic complex also called paired-end complex (PEC), (iii) catalyzes the excision of the element out of its donor site, and (iv) integrates the excised transposon into a new location in target DNA. The majority of known transposases and retroviral integrases contain a highly conserved triad of amino acids, known as the aspartate-aspartate-glutamate, in short, the DDE (or a variant of it composed of DDD), signature in their C-terminal catalytic domains [1,2]. These amino acids play an essential role by coordinating, in general, two Mg^++^ ions required for the catalytic steps (DNA cleavage and joining) of transposition [3,4].

The key biochemical process of all transposon excision reactions executed by DDE recombinases is the exposure (by a single-strand nick) of 3′-OH groups at the transposon ends, which will later be used at the strand transfer reaction for integration [5]. During cut-and-paste transposition, nicking of the element is followed by the cleavage of the complementary DNA strand resulting in a double-strand break (DSB) that liberates the transposon from the donor DNA. To catalyze second-strand cleavage, DDE recombinases developed versatile strategies [6]. Most DDE transposases cleave both strands of DNA at the transposon end via a DNA–hairpin intermediate. For example, in transposition of the *hAT* superfamily element *Hermes* [7] and *Transib* [8], the single-strand nick is converted into a DSB by a transesterification reaction, in which the free 3′-OH attacks the opposite strand, thereby creating a hairpin intermediate at the donor site (reviewed in [6]). The bacterial Tn*5* and Tn*10* transposons and the insect *piggyBac* (PB) element also transpose via a hairpin intermediate, with the difference that the hairpin is on the transposon ends and not on the flanking DNA [9,10,11]. For the transposition reaction of these elements to proceed, the hairpin must be opened by a second hydrolysis to expose a 3′-OH group at the transposon end, which will be used for strand transfer during integration. Members of the Tc1/*mariner* family do not transpose via a hairpin intermediate [12,13], indicating that double-strand cleavage is the result of two sequential hydrolysis reactions by the transposase [14,15].

Strand cleavage can occur at different positions relative to the transposon ends. The position of 5′-cleavage of the second strand required for the liberation of the element occurs directly opposite the 3′-cleavage site for the bacterial Tn*5* [16] and Tn*10* elements [10], thereby generating blunt-ended products. In the case of the Tc1*/mariner* elements, the non-transferred strand is cleaved a few nucleotides within the transposon [13,17,18,19], thereby generating 3′-overhangs, whereas *hAT* superfamily transposons excise either blunt-ended or with a one-nucleotide 5′-overhang [7,8]. The prominent pathway of repairing transposon excision sites is nonhomologous end-joining (NHEJ), which generates transposon “footprints” which arise from the transposon-specific overhangs created by transposon excision. In addition to the footprints determined by canonical transposon excision, both hairpin resolution and indels introduced by the error-prone NHEJ repair process contribute to sequence heterogeneity at transposon excision sites [13]. The second step of the transposition reaction is the transfer of the exposed 3′-OH transposon tip to the target DNA molecule by transesterification. Concerted nucleophilic attack at staggered positions in the two strands of the target DNA results in characteristic target site duplications (TSDs) whose sequence reflects the specific target site preferences of every transposon family. At the primary DNA sequence level, integration site preferences are dictated by protein-DNA interactions of the transposase proteins. On the genomic level, the insertion distribution of most transposons is nonrandom, primarily dictated by protein—protein interactions between the transposase and chromatin-bound host factors. 

In nature, DNA transposons exist as single, mobile units where the transposase coding sequences are situated between the TIRs. However, nonautonomous transposons that are unable to express a functional transposase can still be mobilized by transposases expressed from distinct elements in the same genome. Thus, under laboratory conditions, it is possible to use transposons as gene delivery systems, in which virtually any DNA sequence of interest can be placed between the transposon TIRs and mobilized by trans-supplementing the transposase (Figure 1) in form of an expression plasmid or mRNA synthesized in vitro or recombinant protein expressed in *E. coli*. This feature makes transposons natural and easily controllable DNA delivery vehicles that can be used as tools for versatile applications ranging from somatic and germline transgenesis to functional genomics and gene therapy. 

This article provides an overview of the three most widely used transposon systems for genetic engineering in vertebrate animals, including humans. We also review the most important areas of applications of these systems as well as their relative advantages and disadvantages.

## 2. The *Sleeping Beauty* Transposon System

*Sleeping Beauty* (SB) is a synthetic element derived from inactive copies of a DNA transposon of the Tc1/*mariner* superfamily that was at its prime approximately 10 million years ago [20]. It was awakened from its sleep by Ivics et al. in 1997 [21] via the construction of a consensus sequence from eight different fish species by elimination of the inactivating mutations. Following reconstruction, SB was also found capable of mediating transposition in a variety of vertebrates including mice and humans [21,22], and hence, it progressively stood up as a practical tool for genome engineering with applications from functional genomics [23] to gene and cell therapy [24].

**Figure 2:**
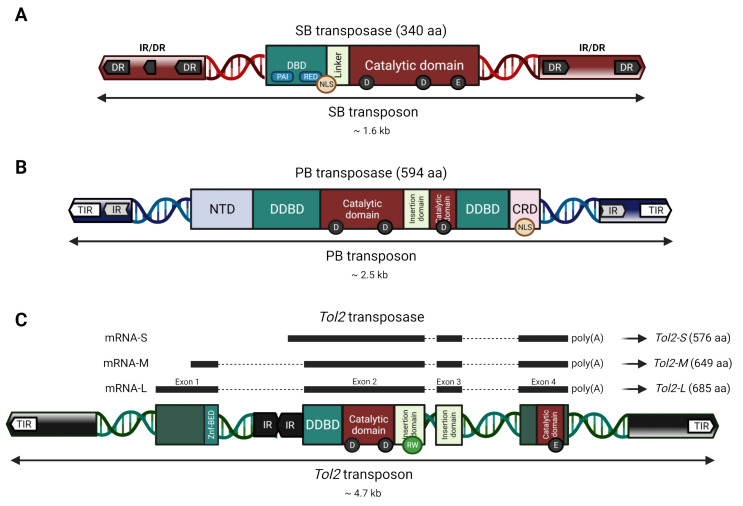
Organization and functional domains of the *Sleeping Beauty* (SB), *piggyBac* (PB), and *Tol2* autonomous transposable elements and transposases. Transposons are depicted as a double-stranded DNA helix flanked by TIRs (arrows). Transposases within each autonomous transposon appear with their respective protein domains (rectangles) after transcription and translation. (**A**) The SB transposon is flanked by TIRs in an inverted repeat/direct repeat (IR/DR) structure (dark grey and red arrows). The SB transposase is depicted with its domains, including a nuclear localization signal (NLS, orange circle), and the PAI and RED subdomains (blue rounded rectangles) of the DNA binding domain (DBD, green rectangle). (**B**) The PB transposon is flanked by its TIRs and subterminal IRs (white, blue and light grey arrows). The PB transposase is shown with its domains and NLS (orange circle). (**C**) The *Tol2* transposon is flanked by its TIRs and subterminal regions (white and black arrows). The autonomous *Tol2* transposon contains an internal *Angel* element (IR black arrows) and the *Tol2* transposase coding sequence with its four exons coding for different protein isoforms (black bars). *Tol2* is shown with its domains, as well as the typical RW-motif (light green circle) of the members of the *hAT* family. The structure of *Tol2*’s putative functional domains was matched with the coding sequence based on the general domains of the *hAT* family members [28], the nucleotide positions on the *Tol2* DNA sequence (DDBJ/EMBL/GenBank accession no. D84375), and previous analysis of *Tol2* [29,30,31]. NTD: N-terminal domain; CRD: Cysteine-rich domain; DDBD: Dimerization and DNA-binding domain; Znf-BED: BED-type zinc finger domain.

The SB transposon is a genetic element flanked by two TIRs of approx. 230 bp arranged in a so-called inverted repeat/direct repeat (IR/DR) structure with inner and outer DRs (Figure 2A). The DRs are at the center of the interactions with the SB transposase, and are therefore critical for transposition; however, the surrounding sequences and spacing between the DRs also play an essential role [25,26,27]. The SB transposase is a 340 amino acid (aa) protein composed of an N-terminal DNA-binding domain (DBD) (aa 1–109) with two subdomains called PAI and RED, followed by a flexible interdomain linker (aa 110–127) carrying a nuclear localization signal (NLS) that overlaps with the DBD (aa 104–120) and a C-terminal catalytic domain (aa 128–340) harboring the DDE triad (D153, D244, E279) [21] (Figure 2A).

The SB transposase orchestrates the transposition reaction in a particularly organized manner (reviewed in [32]). The PAI subdomains play a major role in DNA binding, and lead the assembly of the PEC by recognizing the common core sequences of the DRs [33,34]. The RED subdomains primarily associate with the inner DRs, and trigger dimerization of transposase molecules [35]. Two additional SB transposases finally complete the assembly of the PEC as they bring in the outer DRs and form an SB transposase tetramer [33]. Excision of the SB transposable element only occurs after the formation of the entire synaptic complex. The excision site is primarily repaired by NHEJ, thereby leaving behind a characteristic CAG footprint originating from the terminal sequences of the SB transposon that remain at the excision site after cleavage by the transposase [13]. SB transposon integration occurs predominantly at TA dinucleotides [36] and, due to the staggered positions of the transesterification reactions by the transposon ends [21], the integrated element will be flanked by characteristic TA TSDs [13].

In contrast to other integrative vectors, SB shows a close-to-random integration profile in mammalian genomes [37,38,39], a property of particular interest in the clinical context to lower the risks of insertional mutagenesis. In addition, there are no known endogenous proteins derived from SB in the human genome that could have sufficient structural similarities to interact with the TIRs and mobilize the SB transposable element. Finally, transposases could in theory recognize sequences similar to the TIRs in the genome to mobilize genomic DNA; however, the remarkably organized and complex DNA binding mechanisms required before cleavage act as regulatory checkpoints and ensure high levels of specificity compared to transposon systems with simple TIRs [27,40,41]. Overall, the SB transposon system shows a particularly favorable safety profile and is considered safer than other available integrative vectors for therapeutic gene delivery.

Enhancing the binding affinities of the SB transposon with its transposase is possible by modifying the TIRs but has been somewhat challenging as enhancing one step can impair the order of the assembly [27,35]. The first generation of transposon vectors (pT) underwent mutations in the right TIR and on the nucleotides flanking the transposon ends to give rise to a second generation of vectors (pT2) with a four-fold increase in transposition efficiency [25]. The discovery of a half-DR within the left TIR that acts as a transpositional enhancer [33] (Figure 2A) led to a third generation of vectors (pT3), with a duplication of the left TIR resulting in a two-three-3 fold increase in efficacy [42]. Mutations in the PAI interaction motif of the inner DRs then optimized the binding affinity of the transposase and led to a fourth generation (pT4), with a two-fold increase in activity compared to pT2 [35]. Finally, the addition of IR/DRs disabled for cleavage in a sandwich configuration (pT/SA) was shown to enable a three-fold more efficient mobilization of large transgenes (>7 kb) [26].

Hyperactive SB transposases have also been engineered to enhance the integration of genetic cargo. The first-generation SB10 transposase [21] underwent several modifications based on phylogenetic comparison with related transposases, alanine scans, codon optimization, and rational aa substitutions that resulted in up to 17-fold higher efficiency [43]. In 2009, a high-throughput genetic screen shuffling the previous set of mutations and other phylogenetically conserved aa, followed by successive rounds of manual combinations gave birth to SB100X [44], a hyperactive variant with 9 aa that differentiates it from SB10. These mutations are believed to affect the folding of the protein [44,45], and result in an approximately 100-fold higher transposition activity compared to SB10, providing an integration efficiency comparable to retroviral vectors. The availability of the crystal structure of the catalytic domain [45], the NMR structures of the PAI [34] and RED subdomains [46], and predictions of the tertiary structure of SB100X [47] should enable even further engineering by rational design. An in silico strategy already yielded an SB transposase mutant (I212S), named hySB100X, with 30% higher transposition activity compared to SB100X [45]. A structure-based approach also helped to generate a K248T integration-deficient transposase with highly preserved excision activity and precise footprint generation [48]. Finally, another in silico approach produced a C176S/I212S high-solubility SB transposase (hsSB) with enhanced solubility and stability for delivery as a recombinant protein [49]. This engineered variant showed even spontaneous penetration within cells without requiring transfection or electroporation techniques (see *Vectors* section).

SB supports a full spectrum of genetic engineering applications/methods (reviewed in [50]), including the generation of transgenic cell lines, induced pluripotent stem cell (iPSC) reprogramming [51,52,53,54,55,56], phenotype-driven insertional mutagenesis screens in the area of cancer biology (reviewed in [23,57,58]), germline gene transfer in experimental animals [59,60,61,62,63,64,65], and somatic gene therapy both ex vivo and in vivo (reviewed in [24,44,50,66,67,68,69,70,71]), which will be discussed in the following sections of this review.

## 3. The *piggyBac* Transposon System

It was recognized in the early 1970s that certain baculovirus occlusion bodies changed their morphology when passaged in cell lines derived from the cabbage looper moth (*Trichoplusia ni*) [72]. This phenotype was identified to result from host cell DNA insertions into the virus genome disrupting viral genes, which could be reverted and DNA insertions lost by continuing passaging in the same cell line [73]. Based on the observation that sequences homologous to the insertions found in the baculovirus genome were represented in multiple copies across the *T. ni* genome, it was strongly suspected that the underlying mechanism is that of an active mobile DNA element jumping from insect to virus genome. This was ultimately confirmed, with the full transposable element sequence described in 1989 (at the time called IFP2) [74]. Almost a decade later, the responsible protein mediating transposition was identified, found to be capable of excision and integration of nonautonomous DNA elements flanked by IFP2 termini [75], and thus, giving birth to the PB transposon system.

The original PB transposon has a total length of 2475 bp [74], and harbors a single open reading frame (ORF) encoding a transposase. The transposase itself is 594 aa long, and contains dimerization and DNA-binding domains, a catalytic domain including the DDD catalytic triad (positions 268, 346, and 447) interrupted by an insertion domain, an acidic N-terminal domain and cysteine-rich C-terminal domain (CRD) [76], the latter of which includes an NLS predicted to span from positions 551 to 571 [77] (Figure 2B). The ORF is flanked by terminal sequences containing asymmetric inverted repeats: 13-bp long TIRs separated, by 3-bp in the left and 31-bp in the right terminal sequence, from 19-bp long sub-terminal repeats critical for transposon end recognition and target for CRD binding [78], respectively.

Despite its origin in insects, the activity of PB is not limited to this class and PB has been shown to be active in vitro [11], as well as in a wide variety of organisms comprising yeast [11], plants [79], and mammals [80], including human [81]. As with other transposon systems, the efficiency of PB transposition displays a negative correlation with transposon size. Nonetheless, several studies have shown the possibility to use the PB transposon system for bacterial artificial chromosome (BAC) transgenesis, mobilizing giant transposons over 100 kb in length maintaining cargo integrity [82,83].

In contrast to SB’s preference to integrate into TA dinucleotides, PB almost exclusively integrates into TTAA sequences [84], which are duplicated and flank the inserted transposon after integration. A distinctive and highly attractive characteristic of the PB transposase is that, in contrast to most transposases, its excision leaves no footprint behind at the excision site [74,84]. This plays only a minor role for most applications since transgenes are usually intended to remain integrated. However, as long as active transposase remains in the cell, genomically integrated transposons can be recognized and remobilized to new sites in the genome, and by doing so, footprints arising at the transposon excision sites could produce unwanted effects including frame-shift mutations. As PB transposition rarely produces footprints [85], genomic integrity is minimally compromised after remobilization and allows for prolonged transposase expression. Additionally, the invention of an excision-only PB transposase mutant [86] enabled novel transposon-based applications such as efficient transient transgenesis, e.g., for the generation of iPSCs and seamless removal of reprogramming factors. On a genome-wide level, PB displays a preference for integration near transcriptional start sites (TSSs), similar to γ-retroviruses [38]. A mechanism that provides a likely explanation to this observation is a physical interaction of the PB transposase with bromodomain and extraterminal domain (BET) proteins [38] that bind to acetylated histone tails [87], regulating chromatin structure. Interaction of the murine leukemia virus (MLV) retroviral integrase with BET proteins provides a tethering mechanism resulting in retroviral integration close to the chromosomal regions enriched in acetylated histones including TSSs, and disruption of this interaction using small-molecule BET inhibitors led to a more unbiased insertion site profile [88,89]. Although this phenomenon would have an evolutionary explanation by directing transposition into euchromatin to facilitate transposase transcription, it increases the risk of disrupting transcriptional regulation units of critical endogenous genes. When envisioning potential gene therapy applications, this represents a safety concern [38]. However, a risk of genomic integration always needs to be evaluated in the context of a particular cell type and a particular disease, suggesting the feasibility of clinical applications of PB, for example in CAR-T therapies [90] (see section *Clinical Applications*).

Interestingly, in a process known as “domestication”, transposons are recruited by host genomes and repurposed for useful yet often unknown functions throughout the course of evolution. An example of domesticated PB transposase-derived genes in the human genome, namely PGBD5 [91], codes for a protein which, surprisingly, showed a cross-reaction in human cell culture experiments with insect PB transposons and catalyzed transposition [92]. This could have potential detrimental implications for human applications, as these experiments imply that endogenous human transposases could in principle mobilize exogenous transposon sequences delivered in a gene therapy setting. However, no such events have been observed to this day, presumably due to the endogenous expression levels of PGBD5 being fairly low compared to the levels induced in cell culture experiments and restricted to early embryo developmental stages and brain [91].

The PB transposon system has undergone several modifications and optimizations designed to improve its efficiency: (i) TIR sequences have been adapted to a minimal length required for transposition [93], and sequence-optimized [94] to increase transposition efficiency, (ii) codon usage has been adapted for mouse [95] and human [94], resulting in higher transposition activity in corresponding cells compared to the original sequence of insect origin, and (iii) multiple hyperactive PB transposase mutants have been described [85,96] that significantly exceed efficiencies of the wild-type variant.

Overall, the PB transposon system is a highly recognized tool for genetic engineering. Today, applications range from basic research applications such as mutagenesis screens to discover cancer-driving genes or the introduction of reprogramming factors into somatic cells to generate iPSCs, to gene therapy approaches in preclinical and clinical settings. These will be discussed in the following sections of this review.

## 4. The *Tol2* Transposon System

The *Tol2* transposon element was discovered in 1996 in the Japanese medaka fish (*Oryzias latipes*) [29] by virtue of an albino phenotype caused by an insertion of about 4.7 kb of DNA in the tyrosinase gene. The insertion had structural properties of a DNA-based transposable element and was named “Transposable element of *Oryzias latipes*, number 2” (*Tol2*) and the particular copy in the tyrosinase gene *Tol2*-tyr [29]. This was the first report of an active DNA-based element in vertebrates.

*Tol2* contains imperfect TIRs of 17 bp and 19 bp, and proximal to the right TIR three subterminal repeats of about 30 bp are located. *Tol2* contains three introns and four exons encoding 117, 352, 102, and 118 aa that makes up an ORF encoding a transposase with sequence resemblance [30] to members of the *hAT* superfamily [97], including *hobo* of *Drosophila* [98], *Ac* of maize [99] and *Tam3* [100] of snapdragon. Due to the four exons of the *Tol2* mRNA, several isoforms of transposases exist where the most active variant is the 649 aa isoform [101]. *Tol2* shares a wide range of characteristics with the other *hAT* members, including a catalytic DDE triad (D192, D258, and E611 in mRNA-M) [97,102,103]. *Tol2* additionally contains the RW-motif, which is typically found near the N-terminal end of the insertion domain in members of the *hAT* superfamily [28,103]. Other important functional domains are the dimerization and DNA-binding domain (DDBD) and the BED (which was named after the *Drosophila* proteins BEAF and DREF) zinc finger (znf-BED) [28] (Figure 2C). By studying the different mRNA products transcribed from the *Tol2* element, mRNA-S (short, exon 2–4) was revealed to have an inhibitory effect on excision. Its deduced protein lacks 109 aa of the N-terminal region compared with the functional transposase encoded by mRNA-L (long, exon 1–4). This suggests that the transcript of mRNA-S may act to inhibit the transposition of *Tol2* in medaka fish cells [104].

In medaka and zebrafish, the excision of the *Tol2* transposon is either precise or imprecise [29,105], although it is likely that “imprecision” is a manifestation of hairpin opening and indels generated by NHEJ [106] (as discussed above), rather than an intrinsic feature of the excision reaction itself. On the level of primary DNA sequence, *Tol2* target site selection is considered promiscuous [107], with a weak consensus palindromic-like TNA(C/G)TTATAA(G/C)TNA octanucleotide sequence [101,108]. *Tol2* generates 8-bp target site TSDs at the integration sites. A preference for AT-rich DNA for integration [101] may indicate a preference for incorporation into regions of higher DNA flexibility. *Tol2* transposon integration is biased for DNaseI hypersensitivity sites and CpG islands with a preference toward integrating close to TSSs and transcriptional regulatory regions [107,109,110].

*Tol2* has a fairly large cargo capacity; it can deliver a total of around 200 kb in several mammalian cell types and 10–11 kb without compromising the transposition efficiency [111,112,113,114]. Transgenes cloned into *Tol2* vectors are reliably expressed in transgenic animals and cells. This may suggest that they are free from a putative gene silencing mechanism by the host, which may sometimes suppress the expression of transgenes created by the introduction of plasmid DNA into cells [115]. Improvements of the efficacy of the *Tol2* system include the codon-optimization for enhancement of transposase expression in mammalian systems, including mouse and human [101,116,117]. Epitope-tagged *Tol2* transposase protein (His-*Tol2*) purified from *E. coli* has been shown to maintain high transposition activity in zebrafish embryos. Purified His-*Tol2* was also shown to be capable of performing both the excision and integration steps of transposition in vitro in the absence of any cellular co-factors. The His-*Tol2* protein is a promising new transposase source for molecular medicine and genome engineering applications [108]. By truncating the original *Tol2* to carry a 200-bp 5′-end and a 150-bp 3′-end, the overall size of the *Tol2* element was minimized without compromising the transposition capacity. This “minimal *Tol2*” or mini*Tol2* resulted in a ~three-fold increase in transposition activity when compared to the full-length *Tol2* system [116].

*Tol2* can be used in the generation of transgenic animals (mouse, chicken, frog, and zebrafish models) and insertional mutagenesis (see sections below), and is thereby a suitable vector for gene therapy, since it enables sustained transgene expression after gene delivery [118]. The *Tol2* transposon system-based gene trap approach has helped identify novel developmental genes and novel structures of known developmental genes. Indeed, *Tol2* is widely used in zebrafish, and many transgenic fish lines have been generated through several large-scale genetic screens based on it.

## 5. Vectors for Enhanced Delivery of Transposon Systems

**Figure 3:**
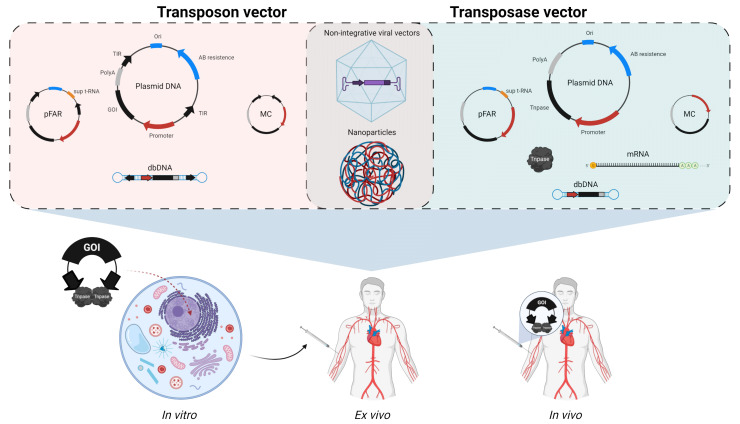
Overview of transposon system delivery methods. The transposon can be delivered as plasmid DNA, plasmid-free of antibiotic resistance markers (pFAR), minicircle (MC), or doggybone DNA (dbDNA) (upper left). The transposase can be delivered as plasmid DNA, pFAR, MC, dbDNA, mRNA, or recombinant protein (upper right). Additionally, hybrid delivery methods combining transposon system components with non-integrative viral vectors or nanoparticles are possible (upper middle). Combined, these delivery methods enable in vitro, ex vivo and in vivo administration (bottom).

The double plasmid setup (an expression plasmid for the respective transposase and a plasmid harboring the gene of interest, flanked by the corresponding TIRs specific to each transposon system) is generally used to deliver the transposon and transposase (Figure 3), as it is the most simple and cost-efficient. Nonetheless, the presence of cytosolic DNA [119], in particular unmethylated CpG dinucleotides from bacterial DNA [120], may trigger immune responses via activation of intracellular DNA sensors and can compromise efficiency and safety of a genome engineering strategy. The presence of antibiotic resistance genes in plasmid DNA may also trigger immune responses and presents risks of dissemination into pathogenic bacterial strains so their use has been discouraged by regulatory agencies for therapeutic applications [121]. In addition, delivering the transposase as DNA implies extended protein expression, and enables only poor control over transposition activity [122], as it may fluctuate depending on transcriptional and translational rates. Last but not least, the delivery as DNA also implies risks of spontaneous genomic integration of the transposase-encoding sequence [123] that could lead to constant expression of the transposase, persistent remobilization of the transposable elements and mutagenesis of endogenous genes by genome-wide footprints [124].

Since nucleic acids do not possess the innate capacity to penetrate and enter target cells by themselves as viral vectors do, external assistance is needed. However, this assistance is straightforward, and well-established protocols exist. For routine work with standard, easy-to-transfect laboratory cell lines, transfections with typical transfection reagents provide a fast and efficient way of enabling transposition reactions [44,85,112]. Additionally, for clinically relevant cell types and non-dividing cells, electroporation techniques such as nucleofection have proven to be an effective way to counter transfection difficulties in challenging cell types [39,125]. Most clinical trials that have been conducted to this day employ nucleofection techniques to shuttle the therapeutic gene cassettes alongside the transposase into human cells. In this scenario, cells are extracted from patients or donors, nucleofected and expanded ex vivo, and undergo quality control before being infused into the patient [126]. Currently, no in vivo administration protocol for transposon systems meets the standards required for human use concerning safety and efficiency. However, alternative delivery methods besides the preferred ex vivo modification are actively being investigated. The following chapters will describe the most relevant delivery methods that have been successfully used in basic research, as well as preclinical and clinical settings. These are not limited to nucleic acids but include recombinant protein and hybrid systems consisting of viral particles or synthetic nanoparticles combined with transposon system-associated nucleic acids, allowing for in vitro, ex vivo, and in vivo systemic delivery.

### 5.1. Minimized DNA Vectors

With the growing interest in non-viral alternatives for genome engineering, the issue of toxic effects caused by the nature of plasmid DNA has been addressed. Plasmids free of antibiotic resistance genes (pFAR) (Figure 3) can be produced from engineered *E. coli* that bear a nonsense mutation in the essential *thyA* thymidylate synthase gene [127]. pFAR vectors encoding a nonsense suppressor tRNA are then able to alter the reading and restore bacterial growth. In addition to avoiding the presence of antibiotic resistance genes, pFARs greatly reduce the overall length of the construct and have shown lower toxicities and efficient transgene delivery in association with the SB transposon system [128,129]. DNA minicircles (MCs) (Figure 3) enable to further reduce the length of the vector as their production permits the removal of the bacterial origin of replication by site-specific recombination [130]. As most of the backbone sequences are removed from the parental plasmid, a transposon vector in an MC can have its TIRs in very close proximity (about 200 bp), a setup that has been shown to enhance transposition efficiency by aiding synaptic complex formation [22,83,131,132]. Therefore, MC vectors have been used in combination with the SB transposon system, and have resulted in decreased cytotoxicity and higher efficiency following electroporation (up to five-fold comparing to plasmid DNA) [39,131]. For the PB transposon system, the delivery of the transgene in form of doggybone DNA (dbDNA) [133] (Figure 3) is an attractive alternative to the MC delivery approach [125]. In contrast to MCs, dbDNA amplification is an exclusively in vitro process except for the propagation of the initial parental plasmid, and thus, presents the added benefit of eliminating the need for endotoxin removal for clinical-grade manufacturing.

### 5.2. mRNA and Proteins

Transposases can be delivered as in vitro-transcribed messenger RNA (mRNA) (Figure 3) to enable only short-term expression, reduce toxicity and avoid risks of chromosomal integration. *Tol2* has been the first transposase to be supplied as mRNA to test its function in zebrafish [31]. PB [134] and SB [135] transposases followed, and studies have shown that mRNA can be used for transposition in vitro and in vivo with high gene transfer efficiency and reduced toxicity [135,136,137,138]. The latest technologies, such as Stabilized Non-Immunogenic Messenger RNA (SNIM-RNA) currently being used with SB100X [39], involve mRNAs bearing chemical modifications for increased stability and lower activation of the innate immune response associated with in vitro-transcribed mRNA [139]. Direct protein delivery (Figure 3) would enable further control over transposase expression, but hurdles in recombinant protein production, purification and delivery into cells have been major setbacks for this strategy. Studies with SB [140] and PB [141] transposases linked to cell-penetrating peptides (CPPs) have, however, provided proofs-of-concept that transposases could be delivered as proteins and penetrate cellular membranes along with the transposon, albeit with limited efficacy. Promising results were obtained with a histidine-tagged *Tol2* (His-*Tol2*) transposase that showed advantages over mRNA in zebrafish to generate transgenic animals with low mosaicism and high germline transmission rates [101]. Finally, a recent study used an in silico strategy based on the structure of SB100X to engineer hsSB, a transposase mutant with enhanced stability and solubility for facilitated protein production and delivery [49]. hsSB was used to mediate transgenesis in diverse mammalian cells, stem cells, and to generate CD19 CAR-T cells with efficiencies comparable to MC-SB100X. Surprisingly, hsSB was even found to be able to spontaneously penetrate cells without requiring transfection or electroporation techniques [49]. This method was used to engineer iPSCs, generate CAR-T cells, and opens new opportunities for engineering other, particularly sensitive cell types.

### 5.3. Hybrid Systems Relying on Non-Integrative Viral Vectors and Nanoparticles

As it relies on natural infectious mechanisms, viral transduction is an efficient method to deliver genetic material into cells and the nucleus. By combining the entry properties of viral vectors and the integrative properties of DNA transposons, hybrid viral-transposon vectors (Figure 3) can this way maintain stable long-term expression while enabling the reduction of the viral load. Recombinant adeno-associated viral vectors (rAAVs) have been successfully used with the PB [142,143,144,145,146] and SB [147] transposon systems to enable integration of the DNA material while taking advantage of AAV tropism towards specific tissues for in vivo delivery. PB [146,148] and SB [147,149,150,151,152,153,154] have also been used with adenoviral (AdV) vectors, baculovirus expression vectors (BEVs), and herpes simplex virus type-1 (HSV) vectors to benefit from their large payload capacity (up to 36 kb, 50 kb, and 130 kb, respectively) and their respective intrinsic properties, namely AdV’s broad tropism, BEV’s low cytotoxicity, and HSV’s preference for the central nervous system. Integration-deficient lentiviral vectors (IDLVs) have also been used for their low immunogenic properties and combined with SB [37,147] to rescue their integration capacity, albeit with a close-to-random profile for improved biosafety as compared to integration-competent lentiviral vectors that target actively expressed genes for integration. An interesting approach that overcomes the protein production and purification issues relies on fusing a hyperactive PB transposase (hyPB) with the lentiviral integrase to enable its packaging within lentivirus-derived nanoparticles (LNPs) and results in effective delivery in human cells [155]. Finally, nanoparticles (Figure 3) are emerging as promising tools for fully non-viral in vivo delivery approaches with ideally low restrictions of cargo capacity, low immunogenicity, and low costs. In fact, strategies using nanoparticles coated with peptides enhancing endosomal escape, containing microtubule-associated sequences, NLS sequences, or targeting specific cell types have shown efficient packaging of the SB [156,157] and PB [158] transposon systems and successful in vivo delivery in preclinical gene therapy settings (see *Preclinical* section below).

## 6. Basic Research Applications

### 6.1. Insertional Mutagenesis Screens

Due to their genome-wide insertion patterns, DNA transposons are by nature mutagenic elements that can impact the function of other DNA features across the genome. This property has enabled them to affect the genome of their hosts for millions of years of evolution and now enables us to exploit them to uncover the hidden features of the genome via forward genetic approaches. Reverse genetic approaches examine the functional consequences induced by specific mutations, a useful strategy for rationale-guided analysis. In contrast, forward genetic approaches rely on screens that enable the identification of the genetic causes of a given phenotype and are therefore more prone to find unknown features within the genome or unknown functions of these features. Transposon-mediated forward genetic approaches are based on high-throughput insertional mutagenesis screens that detect loss-of-function or gain-of-function mutations (reviewed in [23]). For this application, reporter cassettes and specific mutagenic cassettes are incorporated into the transposable element for each specific use (gene trapping, poly(A) trapping, oncogene trapping, or promoter/enhancer trapping). Insertion sites can be recovered by PCR, mapped onto the genome by sequencing, and analyzed to identify the insertions that had a positive or a negative selective impact during the experiment. Such screens can generate large numbers of genome-wide mutations with minimum cost and effort; however, a saturation of a genome requires high transposition activity, and the outcome of the experiment will highly depend on the insertion profile of the transposon system used. Therefore, choosing the right system for the right application is essential. For example, the preferential integration of *Tol2* and PB into TSSs is advantageous for promoter/enhancer trapping [108,159], while the close-to-random insertion profile of SB enables to perform highly unbiased screens and detection of features that would be unlikely reached with other methods [160]. As discussed in previous sections, PB was shown to be highly active in a variety of species from yeast to humans, a property that makes it an almost universal tool for mutagenesis screens [161,162]. SB and *Tol2* have shown to be active only in vertebrates, with *Tol2* generally presenting lower transposition efficiencies, except for the zebrafish germ lineage [163]. Zebrafish are a model of particular interest for research purposes, as the body of their larvae is transparent throughout their entire development [164]. *Tol2* enabled mutagenic screens in this model [159,163], and remains a major tool in zebrafish research [165]. Transposon-based screens are also currently of particular interest in cancer research to identify oncogenes, drivers of metastasis and predictors of therapy resistance [160]. As high-throughput technologies are increasingly accessible and discoveries require more data and efforts, we predict that transposon-based mutagenic screens will play a major role in unraveling the remaining mysteries present within genomic DNA.

### 6.2. Transgenic Animals

Transgenic animals represent a fast-growing component in biotechnology with valuable applications in biological and medical research, agriculture, and pharmaceutical production among others. The most simple and widely used method to generate transgenic animals is via pronuclear microinjection [166], a process in which DNA is directly injected into pronuclei of fertilized eggs, resulting in permanent incorporation of the introduced sequence into the genome, and thus, eventually, into every cell of the animal that develops from the injected zygote. However, the process is highly unpredictable as it relies on random incorporation of the microinjected DNA. The highest efficiencies are achieved with linearized DNA [167], which often recombine and integrate as tandemly arranged concatamers [168] that (i) can persist through multiple cell divisions as episomal DNA [169] and (ii) are prone to silencing [170]. Both of these phenomena can generate mosaic embryos [171]. In addition, non-homologous recombination can cause severe chromosomal rearrangements that include deletions, duplications and translocations [172,173,174] that can be accompanied by substantial deleterious effects in the modified animal.

Transposon systems have proven to be a valuable solution to this bottleneck, by aiding genomic incorporation of precise monomeric transgene units by transposition from a donor plasmid into the host cell genome. Additionally, rapid but transient transposase expression achieved by administration of mRNA serves the purpose of facilitating transposition at early developmental stages while simultaneously limiting transposition at late stages, thereby preventing mosaicism. Importantly, it also circumvents the risks associated with transposase gene incorporation, which could lead to permanent transposase expression and transgene remobilization, as mRNA is unlikely to undergo genomic integration. Interestingly, through a poorly understood mechanism, transgenesis can be achieved quite efficiently by microinjection into the cytoplasm of fertilized eggs; a procedure that barely produces any integration events with linear or circular DNA without a transposase source [167]. This is particularly useful for species in which pronuclei are opaque and hardly visible under the microscope. All three transposon systems outlined in this review have been used for successful germline transgenesis via microinjection. Transgenic animals generated by this method include zebrafish [114,175,176], mice [80,177,178], rats [61,179], rabbits [60], pigs [59,180], and cattle [181].

### 6.3. iPSC Reprogramming

iPSC technology aims to provide an infinite supply of cells for groundbreaking therapies and to cure diseases that have traditionally been considered incurable [182]. In addition to their direct use for cellular therapies, iPSCs offer elegant platforms for drug screenings and disease modeling in conjunction with a variety of disease backgrounds. In brief, by taking a small tissue sample from the patient, somatic cells are reprogrammed to iPSCs with limitless proliferative ability. In the context of a genetic therapy (i.e., if cells originate from a patient with a disease-causing genetic defect), gene correction is performed in the iPSCs and differentiation directed into the desired precursor cells with the final goal to transplant the cells back into the patient.

Given their efficient gene delivery, the SB and PB transposon systems are attractive tools for somatic cell reprogramming. *Tol2* is also active in mammalian cells, but its function in iPSC generation is still unexplored [183]. The overall efficiency of transposon-mediated cellular reprogramming is approximately 0.02 %, which is comparable to the initial reprogramming efficiencies obtained by viral vectors [51,54,184]. Transposons have a higher reprogramming efficiency than non-integrative delivery systems such as replicating episomal vectors or MCs [185,186]. SB and PB provide safe and efficient reprogramming of somatic cells into iPSCs by their defined advantages such as their large cargo capacity of up to 100 kb [83]. This is useful for the mobilization of large and complex transgene cassettes with pluripotency factors, namely Oct4, Klf4, Sox2, cMyc (OKSM), Nanog and Lin28 (OKSMNL) [187]. In addition, the excision reaction uniquely associated with cut-and-paste transposition enables removal of reprogramming cassettes from the genome once reprogramming is complete. In this context, excision-proficient but integration-deficient PB [188] and SB [48] transposase mutants provide an additional advantage in stem cell reprogramming. Because the biochemistry of PB excision allows transgene removal without a transposon footprint left behind at the transposon excision site, this allows for seamless removal of transposon-encoded reprogramming cassettes or other functional transgene cassettes from the iPSC genome [48,189]. This helps to overcome the limitations of viral-based reprogramming technologies. Indeed, PB was used for footprint-free correction of gene mutations responsible for epidermolysis bullosa in iPSCs [190]. In addition, in a novel approach using a combination of gene editing by designer nucleases and PB-mediated seamless excision, iPSCs reprogrammed from peripheral blood mononuclear cells of HIV-infected patients were edited to include the naturally occurring 32-bp deletion in the chemokine receptor 5 (CCR5) [191], known to hinder viral entry [192]. After differentiation into immune cells, resistance to CCR5-tropic and to some extent CCR5/CCRX4-dual-tropic HIV-1 infection was observed, providing exciting developments in potential future functional HIV cures. Finally, not only have human somatic cells been successfully reprogrammed by SB and PB to obtain iPSCs, but the range of possible iPSC models has been expanded to the murine model and even large domestic species such as horse, cattle, pig, buffalo, as well as bat and monkey (reviewed in [187]).

## 7. Preclinical Applications

Transposon systems, as outlined in this review, hold promising potential in the field of gene therapy due to their efficient, safe, and relatively simple way of shuttling transgenes into a wide range of clinically relevant cell types. SB and PB transposon systems have been successfully used in multiple preclinical studies tackling rare genetic disorders, including tyrosinemia and diabetes type I, familial hypercholesterolemia, hereditary hyperbilirubinemias, cystic fibrosis, mucopolysaccharidosis, hemophilia A and B, sickle cell disease, epidermolysis bullosa, Duchenne muscular dystrophy (DMD), and Limb-Girdle muscular dystrophies (LGMD) [24,146,190,193,194]. For these diseases, the treatment typically consists of simple complementation of the defective gene, with transposons being mediators of long-term treatment efficacy and potential curative outcome after a single dose. Transposon systems have also enabled preclinical success for the treatment of complex diseases, caused by a combination of genetic and environmental factors. Notably, strategies of SB-mediated integration of RNA interference (RNAi) have shown improvement in mice with idiopathic pulmonary fibrosis [195] by delivery of miR-29, a microRNA involved in fibrogenesis and often downregulated in fibrotic diseases, and with Huntington disease [196] via delivery of siRNAs to downregulate huntingtin expression. Other strategies have used the established pharmacologic and physiologic principles to ameliorate the condition of diseases such as non-familial pulmonary hypertension [197] by SB-mediated delivery of the endothelial nitric oxide synthase (eNOS) gene to restore the physiologic arterial pressure, and age-related macular degeneration (AMD) [128,198,199] by supplementation of the pigment epithelium-derived factor (PEDF) gene to restore the angiogenic/antiangiogenic balance in the retina. Antiangiogenic strategies [200,201,202], as well as promoter-specific suicide gene delivery approaches [203], have also been used with SB and PB as direct cancer therapies.

Currently, preclinical applications focus mostly on indirect cancer therapies via adoptive immunotherapy. The discovery that synthetic chimeric molecules composed of immunoglobulin and T cell receptor components could be introduced to and guide T cells with an antibody-like specificity to preselected targets [204] proved to be a breakthrough in cancer therapy by targeting malignant cells based on cancer-specific surface markers [205]. As of today, there are five FDA approved chimeric antigen receptor (CAR)-T cell products on the market (Novartis’s Kymriah, Gilead’s Yescarta and Tecartus, and Bristol Myers Squibb’s Breyanzi and Abecma), all based on ex vivo lentiviral or γ-retroviral transfer of the CAR gene into T cells. However, transposon systems represent a viable alternative to mediate gene transfer, and have been continuously tested in preclinical CAR-T cell therapy settings. SB pioneered applications with the well-characterized CD19-specific CAR-T cells (reviewed in [24,206]), and was joined by PB [206,207,208] and *Tol2* [113] to demonstrate suppression of B cell lymphoma progression both in vitro and in vivo. The most recent developments include CAR-T cell engineering for alternative targets such as the granulocyte-macrophage colony-stimulating factor receptor (hGMR or CD116) for hematological malignancies [209], the epidermal growth factor receptor (EGFR) as a target for non-small-cell lung carcinoma [210], glypican-3 and EGFRvIII targeting hepatocellular carcinoma [211,212] and membrane-proximal mesothelin (MSLN) epitope-targeting against MSLN-positive solid tumors [213]. Additionally, SB and PB have been used to generate allogenic “off-the-shelf” CAR-natural killer (NK) cells that have shown encouraging results against solid tumors [214,215]. Cytokine-induced killer (CIK) cells, effector lymphocytes displaying a mixed T and NK phenotype with non-HLA-restricted cytotoxicity and minimal alloreactivity, have also been generated with SB to target CD19, CD123, and CD33 [216,217]. Finally, SB was shown successful for T cell receptor (TCR)-T cell engineering [24,218], a strategy that enables to broaden the spectrum of targets as the MHC-TCR interaction also permits recognition of epitopes from intracellular proteins.

Most of the previously cited applications are based on ex vivo cell modification, because systemic administration of transposon systems in vivo has been challenging. Hydrodynamic injection techniques (reviewed in [146]) have scratched the surface by providing stable gene expression in the liver and kidney of mice but they have remained out of clinical reach due to difficulties in upscaling the procedure to humans and hepatotoxic adverse effects following the injection. Alternative strategies involving packaging transposon systems into non-integrative viral vectors (see *Vectors* section) have, however, shown promising results for in vivo delivery. Various groups have combined AAV vectors, lacking the ability to integrate their cargo, with the PB transposon system. Using this strategy, diabetes type I [142], urea cycle defects [143], cystic fibrosis [145], and progressive familial intrahepatic cholestasis type 3 [144] were corrected in pig and mouse models. Other studies on cancer therapies have reported a reduction of tumor growth in mice and increased lifespan with hybrid SB-baculovirus vectors [150,151,152]. One of the most promising strategies is an in vivo gene transduction system based on a hybrid adenovirus/SB vector system [153]. In a recent study, hematopoietic stem and progenitor cells (HSPCs) were mobilized into the peripheral blood in a transgenic mouse model and directly targeted using a helper-dependent hybrid adenovirus (HD-Ad5/35^++^)/SB vector system intravenously injected into the bloodstream [154]. The hybrid vector targets human CD46 (a receptor that is uniformly expressed on HSPCs in these transgenic mice) and permits the stable genetic engineering of HSPCs in vivo. Ultimately, nanoparticles (see *Vectors* section) combined with the SB transposon system have provided expression of Factor VIII for 50 weeks in hemophilia A mice [156] and long-term cystic fibrosis transmembrane conductance regulator (CFTR) transgene expression in the lungs of cystic fibrosis mice [157]. Remarkably, PB delivered through nanocarriers enabled in vivo generation of CAR-T cells with about 20% of circulating T cells displaying CAR expression after 12 days and potent antitumor activity [158].

## 8. Clinical Applications

The straightforward setup and encouraging results in preclinical studies suggested the utility and distinct advantages of transposon-based, non-viral vectors in clinics. This expectation was met in 2011 when SB entered the clinical stage in the first in-human application of a transposon system worldwide to generate CD19-specific CAR-T cells to treat the minimal residual disease of patients with advanced non-Hodgkin lymphoma (NHL) and acute lymphoblastic leukemia (ALL) after hematopoietic stem cell transplantation (HSCT) [147]. Here, SB was successfully used to shuttle a second generation, CD19-specific CAR cassette in a classic double-plasmid delivery setting [219]. Trials resulted in 30-month progression-free rates of 83% for patients who received autologous HSCTs and 12-month progression-free rates of 53% for patients who received allogenic HSCTs. Overall survival rates were 100% for the autologous and 63% for the allogenic HSCT group. Neither transgene integration hotspots nor acute or late toxicities or exacerbation of graft-versus-host disease were observed, providing proof of the cost-effective, safe, and efficacious use of transposon-based engineered cell products and paving the way for following trials (reviewed in [25]). Furthermore, the CARAMBA clinical trial (Phase-I/IIA; EudraCT: 2019-001264-30) investigates the feasibility, safety, and anti-myeloma efficacy of autologous SLAMF7 CAR-T cells. CARAMBA is the first clinical trial relying on SB technology for CAR-T cell manufacturing in Europe, and the first clinical trial that uses advanced SB technology (hyperactive SB100X transposase encoded as synthetic mRNA in conjunction with CAR transposon supplied as MC) worldwide [126].

Hematological malignancies are a particularly suitable target for CAR-T cells due to the availability of specific surface antigens and tolerable on-target, off-cancer toxicity with immune reconstitution therapy. Lack of tumor-specific surface antigens in solid tumors with the added challenge of overcoming often-associated immunosuppressive microenvironments, still represent a major hurdle for the broad adoption of CAR-T cell therapy for most cancer diseases. However, recent advances in next-generation sequencing have enabled rapid and economically viable generation of transcriptome data of tissue samples, including tumors. This allows the detection of aberrant tumor-specific gene expression that can be used to identify suitable neoantigen targets for adoptive immunotherapy (reviewed in [220]). Accordingly, synthetic neoantigen-specific TCRs can be designed and shuttled into T cells, followed by adoptive cell transfer into the patient. Aided by simple yet efficient transgene delivery by SB technology, this approach is currently being evaluated to treat patients with glioblastoma, non-small cell lung cancer, breast cancer, gastrointestinal cancer, genitourinary cancer, and ovarian cancer (NCT04102436). Truly personalized T cell therapy, meaning one product per patient, requires fast and inexpensive manufacturing processes more than ever; a requirement the SB transposon system is capable of fulfilling in contrast to viral vectors [221].

Besides cancer, SB is the first transposon system that has made the jump as a tool in other gene therapy applications. In Alzheimer’s disease, impaired nerve-growth factor (NGF) supply to cholinergic neurons leads to their degeneration [222], correlating with the cognitive decline of affected patients. SB was used to engineer cells within an encapsulated biodelivery device to secrete NGF after implantation directly into the brain [223] (NCT01163825). Additionally, for patients with Hurler syndrome, SB will be used to genetically engineer autologous plasmablast to express α-L-iduronidase when transplanted back into the patient (NCT04284254).

Although the SB transposon system has been established as the preferred choice for clinical applications, entering the clinic a decade ago as the first transposon system ever being used in patients [147] and currently employed in the majority of clinical trials out of any transposon system, PB has recently entered the stage as well. The current focus lies exclusively on adoptive immunotherapy in form of CAR-T cell therapy that was analogous to successful SB-based CAR-T cell trials, a therapeutic expression cassette that is transferred into the genome by PB vectors. A first-in-human Phase-I study conducted in Australia (The CARTELL study, NHMRC identifier: 1102172) introduced PB into the clinics in 2016, used to manufacture CD19-specific CAR-T cells infused to patients suffering from relapsed/refractory CD19^+^ malignancies, namely B-cell ALL and NHL. Preliminary reports suggest similar results as expected with viral-vector generated CD19 CAR-T cells [224]. Two additional clinical trials with centers in Japan (UMIN Clinical Trials registry ID: UMIN000030984) and China (clinicaltrials.gov ID: NCT04289220) are currently being conducted/planned, making use of the PB system as well, to manufacture CD19-specific CAR-T cells to treat CD19^+^ B-cell malignancies. Furthermore, expanding on CAR-T cell targets, Poseida Therapeutics Inc. is sponsoring two US-based clinical trials making use of PB technology to manufacture B-cell maturation antigen (BCMA)-specific CAR-T cells for patients with relapsed/refractory multiple myeloma (clinicaltrials.gov ID: NCT03288493) and prostate-specific membrane antigen (PSMA)-specific CAR-T cells for patients with metastatic castration-resistant prostate cancer (clinicaltrials.gov ID: NCT04249947). Reports of the Phase-I BCMA-specific CAR-T cell trial show highly encouraging results with significant efficacy and low rates of cytokine release syndrome and neurotoxicity [225]. Accordingly, a subsequent Phase-II study has begun with a planned cohort of 100 patients in an outpatient setting given the unique safety profile observed in Phase-I [226].

## 9. Conclusions and Outlook

Transposon-based technologies have enormous potential to develop powerful genomic tools with the vision of creating a bridge between physiology and genetics and establishing safe and inexpensive protocols for clinical gene transfer. Different transposon systems do not function at comparable efficiencies, particularly in the context of primary human cells or ultimately in preclinical and clinical trials. Cell type, transposon/transposase ratio, transfection efficiency, DNA concentration and read-out (i.e., mobilization or rescue, gene expression, and antibiotic resistance) are variables affecting the relative transposition efficiency of these different transposon systems (Table 1). By using the same conditions with rigorous controls and consequent head-to-head comparisons, the optimal transposon system can be identified for the respective individual application. In addition to efficacy, integration site preference can greatly influence the utility of transposon vectors for different applications. For example, human gene therapy protocols require the application of transposon vectors showing the least preference to integrate into genes, for obvious safety reasons. The SB system (that shows close-to-random insertion site distribution) appears to satisfy these needs the best, whereas the PB and *Tol2* systems (that prefer genes and their upstream regulatory regions for insertion) appear to be less favorable for potential therapeutic applications. Unlike in therapeutic applications, hitting genes by insertional elements is the goal with forward mutagenesis screens. However, the insertional biases associated with vector systems represent the main limitation to full genome coverage with individual transposon-based vectors. Thus, in this respect, the utility of transposons for mutagenesis is greatly enhanced by the availability of multiple, alternative vector systems with distinct preferences for insertion.

In this article we described the SB, PB and *Tol2* transposon systems; these are the most widely applied transposons in basic, preclinical, and clinical research and development today. However, other elements have been shown to catalyze efficient transposition in vertebrate model organisms. For example, the insect elements *Minos* [227,228] also catalyzes efficient transposition in mammalian cells. *Minos* was also shown to be active in the basal chordate *Ciona intestinalis* [229]. Moreover, the reconstructed amphibian element *Frog Prince* [230], the reconstructed human *Hsmar1* element [18], the reconstructed zebrafish transposon *Harbinger3_DR* [231] and the *Tol1* [232] element isolated from the medaka fish have been found to be active in vertebrate species. The reconstructed bat transposon *Helraiser*, a member of the *Helitron* superfamily, was recently shown to undergo highly efficient transposition in human cells [233]. *Helitrons* are copy-and-paste replicative transposons, and therefore, the *Helraiser* transposon system may be especially useful for the generation of transgenic cells with high copy numbers of genomically integrated transgenes. Finally, the ZB transposon (Tc1/*mariner* superfamily) has recently been characterized in zebrafish; ZB is highly active and displays effective mutagenesis and enhancer trapping in vertebrates [234].

Viral vectors represent a reliable and well-established toolbox from which to choose when planning transgene delivery for transient as well as long-term expression, due to their innate capacity to transduce various cell types with remarkable efficiency (reviewed in [235]). Arguably, at the forefront of human gene therapy, they do not come without downsides. Namely, virus particle production, handling, and downstream processing is expensive, cumbersome, and requires extensive quality control when intended for human application [236]. The cargo capacity of viral vectors varies greatly, but invariably reaches strict limits [235] due to limitations in packaging capacity. Innate and adaptive immune responses to viral vectors [237] constitute safety risks and a major hurdle for in vivo administration. Finally, regarding current viral vector options for stable transgene insertion, lentiviral and γ-retroviral vectors have an integration preference for transcriptional units and TSSs, respectively, which enhances the potential for insertional mutagenesis and oncogenesis (reviewed in [238]), which are important considerations in a clinical setting. We describe aspects in which transposon systems may provide an edge over viral systems for coming applications.

First, one of the most substantial yet often underestimated advantages that transposon systems provide is the ease of use concerning manufacturing and handling of the components. The most basic and widely used configuration is two-plasmid delivery, and both components include a bacterial backbone usually composed of an antibiotic resistance marker and bacterial origin of replication (Figure 3). This setup enables fast and efficient propagation, as well as manipulation in bacteria using traditional plasmid cloning techniques in any BSL1 laboratory with standard equipment. Alternative ways of delivery, as described in previous sections, allow for a flexible combination of individual components to satisfy different needs. These range from “quick-and-dirty” procedures to clinical settings, where stringent quality control of GMP manufacturing is required. All transposon systems used in clinics benefit from the comparatively inexpensive and simple nature of GMP-grade nucleic acid production. As an example, estimations place the per-patient manufacturing costs for SB transposon system components at about 10% of that of the alternative viral vector option [69].

Second, a physical hull encapsulating their cargo does not exist for and thus does not restrict jumping transposons. The benefit of this is that transposon systems substantially exceed the cargo capacity of viral vectors, enabling mobilization of massive transgenic cassettes [82,83,114]. Considering potential future directions, this could allow the design of novel transgene cassettes containing multiple transcriptional units, each driven by native full-length promoter regions, enabling fine-tuning of individual transgene expression, mimicking endogenous transcription levels and driving complex genetic circuits in an all-in-one package.

Third, in vivo gene transfer benefits from the low immunogenic potential transposon system components represent in contrast to viral particles. This poses a substantial hurdle for systemic administration and limits repeated vector administration through innate immune responses, T cell responses, and neutralizing antibody formation (reviewed in [237]). Nucleic acids alone have been successfully employed for in vivo gene delivery in a variety of tissues and through diverse techniques in the past [239,240] without major immunogenicity issues, but often lack sufficient specificity and efficiency. However, with the latest developments in nanomaterials as carrier systems [241], transposon-based integrating vector components could be packed in functionalized nanocarriers tackling both issues while maintaining a low immunogenic profile for in vivo delivery.

Fourth, safety concerns concerning the problematic insertion profiles of viral vectors are significantly lowered by SB’s close-to-random integration behavior, as risk-associated genomic regions are targeted coincidentally instead of through a biased mechanism, thereby lowering the overall probability of integration into “dangerous” genomic regions. A targeted transposon system would, however, provide additional safety by allowing integrations only into predetermined loci, such as genomic safe harbors. In contrast to transposons, the CRISPR/Cas9 nuclease system can make precisely targeted insertions following homology-dependent repair (HDR) of a site-specific DSB utilizing an exogenously supplied DNA template. HDR is, however, not the favored DNA repair pathway; its frequency is cell type- and cell cycle-dependent and its efficiency drops significantly with increasing insert length [242]. A combination of both CRISPR/Cas9 and transposon systems has been suggested to overcome their respective limitations, the former enabling high specificity and the latter providing highly efficient integrative capacity, especially for large DNA fragments. Rather than competing with each other, both technologies should then complement one another and enable the expansion of the possibilities for genome engineering. Remarkably, Tn*7*-like transposons have been shown to use nuclease-deficient CRISPR/Cas systems for RNA-guided transposition with nearly up to 100% of targeted integrations in bacteria (reviewed in [243]). However, their activity has not yet been reported in vertebrates. A different approach achieved up to 90% of targeted integrations in human cells through Cas9-mediated Homology-Independent Targeted Integration (HITI) of SB transposon vectors after fusion of Cas9 with the N-terminal DBD of the SB transposase [244]. Nonetheless, CRISPR/Cas9-induced DSBs implies risks of deleterious on- and off-target mutagenic events and even complex chromosomal rearrangements [245], safety concerns that would argue in favor of using active transposases rather than active nucleases for integration of genetic material. Both the SB as well as the PB transposon systems have hence been the subject of extensive engineering aiming at accomplishing targeted transposition in human cells. Latest developments show the flexibility of transposases to be fused to catalytically dead Cas9 (dCas9) enzymes which, guided by sgRNAs, tether the transposase and its cargo to their target sequence [246,247]. While targeting efficiency remains low, these studies show the potential of transposon-based systems for even safer transgene integration for clinical applications. In summary, attractive features distinctive to transposon systems have set the stage for them being adopted in numerous basic research and preclinical studies, culminating in clinical trials.

## Figures and Tables

**Table 1:** Comparison of *Sleeping Beauty*, *piggyBac,* and *Tol2* characteristics, associated technologies and applications.

	*Sleeping Beauty* (SB)	*piggyBac* (PB)	*Tol2*
**Species of origin**	Salmonid fish [21]	Cabbage looper moth	Medaka fish [29]
**Classification**	Tc1*/mariner* superfamily [21]	PB superfamily	*hAT* superfamily [30]
**Transposable element**	~1.6 kb long	~2.5 kb long	~4.7 kb long
**Terminal regions**	IR/DRs of ~ 230 bp	35–63 bp with outer TIRs and inner subterminal IRs	150–200 bp containing the TIRs and subterminal regions
**Transposase**	340 aa	594 aa	649 aa (most active isoform)
**Footprint**	CAG [13]	None [84]	Variable [29]
**Target site preference**	TA [36]	TTAA [84]	Weak consensus sequence TNA(C/G)TTATAA(G/C)TNA [101]
**Target site duplication**	TA [13]	TTAA [84]	8 bp [101]
**Activity in species**	Various vertebrates	Vertebrates, insects, plants, yeast	Various vertebrates
**Efficiency in human cells**	Comparable to retroviral vectors [44]	Comparable to retroviral vectors [85]	Lower than PB and SB [107]
**Cargo capacity**	>100 kb [83]	>100 kb [83]	>100 kb [114]
**Overproduction inhibition**	Yes [107]	To some extent [107]	Lower than PB and SB [107]
**Integration profile**	Close-to-random [107]	Biased towards TSSs, CpG islands and DNaseI hypersensitivity sites [107]	Biased towards TSSs, CpG islands and DNaseI hypersensitivity sites [107]
**Most common parental plasmid**	pT2	pXL-BacII	p*Tol2*, mini*Tol2*
**Most hyperactive transposase**	hySB100X [45]	hyPB [85]	h*Tol2*-M [101]
**Vectors for transposon delivery**	Plasmid DNA, pFAR, MC, non-integrative viral vectors, nanoparticles	Plasmid DNA, dbDNA, non-integrative viral vectors, nanoparticles	Plasmid DNA
**Vectors for transposase delivery**	Plasmid DNA, mRNA, SNIM RNA, recombinant protein (hsSB), non-integrative viral vectors, nanoparticles	Plasmid DNA, mRNA, non-integrative viral vectors, nanoparticles	Plasmid DNA, mRNA, recombinant protein (His-*Tol2*)
**Clinical trials**	Yes	Yes	No

## Data Availability

No new data were created or analyzed in this study. Data sharing is not applicable to this article.
